# Differential risk profiles for geriatric depression, anxiety, and sleep disturbances in rural China: insights from the Taierzhuang cohort

**DOI:** 10.3389/fpubh.2026.1756508

**Published:** 2026-05-20

**Authors:** Renfeng Zhang, Qian Yu, Yunshan Wang, Xiaoyi Zhao, Shuyi Yu, Zhongyu Dong, Jinghai Hou, Xingguo Qin, Ming Li, Mo Wang

**Affiliations:** 1 Department of Clinical Laboratory, Shandong Provincial Hospital Affiliated to Shandong First Medical University, Jinan, China; 2 People's Hospital of Taierzhuang District, Zaozhuang, China; 3 Department of Neurology, People's Hospital of Taierzhuang District, Zaozhuang, China; 4 Department of Vascular Surgery, Shandong Provincial Hospital Affiliated to Shandong First Medical University, Jinan, Shandong, China

**Keywords:** anxiety, cohort, depression, geriatric mental health, public health

## Abstract

**Background:**

Mental health disorders among older adults in rural China present substantial public health challenges, yet their differential risk factors and practical screening tools remain understudied.

**Methods:**

We analyzed data from 4,180 participants in the Taierzhuang Aging Cohort. Using multivariate logistic regression, we identified independent risk factors for depression, anxiety, and insomnia. Predictor variables were confirmed to be sufficiently independent by evaluating bivariate correlations, variance inflation factors, and linearity in the logit. Corresponding nomograms were developed for risk prediction.

**Results:**

The prevalence of anxiety symptoms, depressive symptoms, and insomnia was 22.1%, 22.7%, and 29.5%, respectively. Multivariate analysis revealed distinct risk profiles for each condition: the absence of a chronic disease (OR: 1.57; 95% CI: 1.28–1.94) and older age were independent predictors of depressive symptoms; while a poor spousal relationship (OR: 1.55; 95% CI: 1.11–2.16) was a risk factor for anxiety symptoms, while physical activity 1–2 times/week (vs. ≥3 times/week: OR = 0.69; 95% CI: 0.53–0.90) was associated with decreased risk; inconstrast, female gender (OR: 1.33; 95% CI: 1.06–1.67) and lower exercise frequency were significantly associated with insomnia. Based on these findings, we developed three clinical prediction nomograms that integrate these independent predictors to facilitate individualized risk assessment.

**Conclusion:**

This study establishes distinct risk profiles for common mental health conditions in rural Chinese older adults and provides practical nomograms for risk assessment in primary care settings, facilitating targeted prevention strategies for these under-served populations.

## Introduction

The global population is aging at an accelerating pace, with projections indicating that individuals aged 60 years and older will comprise 22% of the global population by 2050 (1). Against this demographic backdrop, safeguarding the mental health of older adults has emerged as a critically urgent public health priority. Anxiety disorders, depression, and sleep disturbances represent highly prevalent mental health conditions affecting this demographic. Evidence indicates that over 20% of older adults experience clinically significant anxiety symptoms, while approximately 7% worldwide suffer from depression (2). These mental health conditions not only substantially diminish emotional well-being and overall quality of life but are also strongly associated with functional decline, increased healthcare utilization, and elevated mortality rates, collectively contributing to a substantial disease burden. Notably, anxiety disorders accounted for 8.21 million disability-adjusted life years (DALYs) among adults aged 55 years and older globally in 2021, representing a 2.5-fold increase since 1990 (3–5).

In China, particularly in rural regions, mental health challenges among older adults present distinctive complexities and heightened severity (6). Systemic factors including relative scarcity of healthcare resources, limited accessibility to specialized mental health services, and the pervasive phenomenon of “left-behind” older adult populations collectively exacerbate vulnerabilities in this demographic. Empirical research confirms that the “left-behind” status itself exerts detrimental effects on psychological well-being (7). Furthermore, insufficient mental health literacy, coupled with persistent stigma and cultural misconceptions surrounding psychological disorders, creates additional barriers to early detection and effective intervention in rural communities (8). Compounding these challenges, depression, anxiety, and sleep disturbances frequently demonstrate intricate interrelationships, often forming self-perpetuating vicious cycles. For instance, depression and anxiety constitute well-established risk factors for sleep disorders, with depressed older adults facing a 3.555-fold increased risk of developing sleep disturbances compared to their non-depressed counterparts (9–11).

Despite growing recognition of these mental health challenges, significant limitations in current research hinder the development of effective prevention and intervention strategies. Epidemiological studies reveal that approximately 50%−60% of individuals with depression concurrently meet diagnostic criteria for anxiety disorders, while insomnia represents one of the most prevalent symptoms across both conditions (12, 13). However, this frequent clinical co-occurrence may obscure fundamental etiological distinctions and has impeded the delineation of disorder-specific risk profiles. Current research approaches remain fragmented, with most studies examining single disorders in isolation or focusing on limited sets of conventional risk factors (14). This methodological limitation has precluded systematic comparisons of risk architectures across multiple mental health conditions within the same population, thereby impeding identification of disorder-specific vs. transdiagnostic risk factors and their potential interactions.

Moreover, a pronounced translation gap exists between research and clinical practice. Most existing risk prediction models rely on complex statistical formulations that require specialized software and analytical expertise, rendering them impractical for implementation by rural primary care providers. This disconnect between methodological sophistication and clinical utility has substantially limited the real-world application of scientific advancements in community settings (15–17).

To address these gaps, this study utilizes the Taierzhuang Aging Cohort to: (1) systematically identify and compare shared and disorder-specific risk factors for depression, anxiety, and insomnia among rural older adults; (2) develop and validate practical nomogram-based prediction models for primary care settings. Our innovative multi-disorder framework integrates rigorous risk assessment tool development with examination of unique rural socioeconomic determinants, ultimately aiming to inform targeted mental health strategies for China's rural aging population.

## Methods

### Study design and participants

This study presents a cross-sectional analysis of the baseline data from the ongoing Taierzhuang Aging Cohort, a community-based prospective study in rural China. The investigation was conducted by a team of healthcare professionals from Shandong Provincial Hospital Group Taierzhuang Hospital. Data collection for this analysis was completed between January 2024 and August 2025 through comprehensive field investigations in rural areas of Taierzhuang region.

The study population comprised older adults aged 55 years and above who had been permanent residents in the rural areas for at least 5 years. We established the following eligibility criteria: inclusion required (1) age ≥55 years; (2) permanent residency in the investigated rural communities; (3) capacity to complete the questionnaire interview and physical measurements; and (4) provision of written informed consent. Exclusion criteria included: (1) severe cognitive impairment (as determined by trained interviewers using standardized assessment protocols during the survey); (2) presence of acute medical conditions or terminal illnesses that would compromise participation; (3) significant hearing or language impairments preventing effective communication; and (4) planned relocation outside the study area.

The study protocol received approval from the Ethics Committee of Shandong Provincial Hospital Affiliated to Shandong First Medical University (Approval No.: NSFC-2022-511). All participants provided written informed consent after receiving comprehensive information about study procedures, potential risks and benefits, and their right to withdraw without affecting regular healthcare services.

For this cross-sectional analysis, we included 4,180 participants who completed the baseline assessment, representing the complete analytical sample for investigating the prevalence and risk factors of mental health conditions in this rural aging population.

### Assessment of covariates

Comprehensive covariate data were collected through face-to-face interviews using standardized questionnaires, physical measurements, and well-validated instruments. Trained research staff administered all assessments following established protocols to ensure data quality and consistency across all study sites. The assessment framework encompassed four major domains previously established in geriatric epidemiological research.

Sociodemographic characteristics included age (calculated from birth date and analyzed both continuously and categorically), gender (male/female), educational attainment (categorized from illiterate to university level based on the Chinese education system), occupational classification (from agricultural workers to government staff), marital status (unmarried/married/divorced/widowed), and poverty status (defined according to local governmental standards). Lifestyle factors were assessed using standardized questions adapted from the China Health and Nutrition Survey, including smoking status (current/never/former with current defined as smoking ≥1 cigarette daily), alcohol consumption patterns (current/never/former with current defined as any alcohol intake ≥1 time weekly), physical activity frequency (categorized from ≥3 sessions weekly to < 1 session monthly), and dietary habits focusing on sweet food consumption frequency (from frequent [≥4 times/week] to never).

Clinical measurements included body mass index (BMI, calculated from objectively measured height and weight using calibrated instruments and categorized according to Chinese adult standards), and chronic disease status (based on self-reported physician-diagnosed conditions verified against medication records where available). Social relationship factors encompassed spousal relationship quality (rated as very good/fair/poor using a validated single-item measure), and spouse migrant work status (yes/no), reflecting the unique contextual factors in rural China. All continuous measures were collected using standardized equipment with regular calibration, while categorical variables employed predefined response options to minimize measurement error and enhance data reliability across the study population.

### Assessment of primary outcomes

We employed standardized, validated instruments to assess the primary outcomes, using established dichotomous cutoffs for case identification. Anxiety symptoms were evaluated using the Chinese version of the Zung Self-Rating Anxiety Scale (SAS) (18). Participants rated 20 items on a 4-point scale regarding symptom frequency over the past week. The raw score was converted to a standard index (raw score × 1.25), and a standard index score ≥ 50 defined clinically significant anxiety.

Depressive symptoms were assessed using the Chinese version of the Center for Epidemiologic Studies Depression Scale (CES-D) supplemented with additional clinical items (19), which has been validated for reliability and validity in Chinese older adult populations and exhibits good psychometric properties for detecting depressive symptoms in rural Chinese samples (20). The 22-item instrument measured depressive affect, somatic symptoms, and interpersonal difficulties over the past week. We applied a CES-D score ≥ 16 as the cutoff for clinically significant depressive symptoms.

Insomnia symptoms were measured using the Chinese version of the Pittsburgh Sleep Quality Index (PSQI) (21). This 18-item instrument assesses sleep quality and disturbances across seven components over a 1-month period. A global PSQI score > 7 defined significant insomnia symptoms.

### Statistical analysis

Analyses were performed using R software (version 4.1.0). Descriptive statistics presented continuous variables as means with standard deviations and categorical variables as frequencies with percentages. Prior to formal group comparisons, core statistical test assumptions were rigorously evaluated for validity: independent *t*-tests (for continuous variables including age and BMI) were verified for normality via the Shapiro–Wilk test (all *p* > 0.05) and homogeneity of variance via Levene's test (all *p* > 0.05); chi-square tests (for categorical variables) were confirmed to meet the assumption of expected cell frequency ≥ 5, with over 95% of cells having an expected frequency > 5 and rare cells with lower frequencies accounting for < 3% of the total, which did not affect test validity. Group comparisons used *t*-tests for continuous variables and chi-square tests for categorical variables. Univariate logistic regression identified factors associated with anxiety, depression, and insomnia. Significant variables (*p* < 0.05) were included in multivariate models using forward selection, a stepwise variable selection method adopted to construct parsimonious and clinically interpretable prediction models by sequentially incorporating statistically significant predictors; this approach minimizes overfitting, ensures the final model only contains the most meaningful independent risk factors, and aligns with the practical application demands of primary care settings for the developed nomograms so as to identify independent risk factors. For multivariate logistic regression, four key assumptions were assessed and satisfied: (1) Linearity in the logit for continuous variables (age, BMI) was confirmed via the Box–Tidwell test (all χ^2^ < 1.05, *p* > 0.30); (2) No multicollinearity was found among predictors, with variance inflation factor (VIF) values ranging from 1.02 to 1.20 (all < 5); (3) Independence of observations was satisfied as participants were recruited from independent rural communities with no data clustering; (4) Adequate sample size was confirmed, with the number of events per outcome variable far exceeding the 10 events per predictor threshold. Results were expressed as odds ratios (ORs) with 95% confidence intervals (CIs). Based on final multivariate models, we constructed three nomograms to visualize prediction models for each condition. All tests were two-sided with *p* < 0.05 considered statistically significant.

## Result

### Study population and baseline characteristics

The analytical cohort consisted of 4,180 older adults from the community-based Taierzhuang study. Standardized screening instruments were used to assess the prevalence of anxiety symptoms, depressive symptoms, and insomnia, which were found to be 22.1% (*n* = 924), 22.7% (*n* = 950), and 29.5% (*n* = 1,234), respectively. Table 1 presents the baseline characteristics of participants with each of these three conditions (anxiety, depression, or insomnia) compared with a single healthy control group (defined as individuals with scores below the clinical thresholds for all three conditions). For detailed characteristics stratified by the presence or absence of each individual mental health condition, refer to Supplementary Table S1.

**Table 1:** Baseline characteristics of participants with depression, anxiety, or insomnia compared with a single healthy control group (sub-threshold for all three clinical measures).

Characteristic	Level	Healthy control (*n* = 1,424)	Anxiety (*n* = 924)	*p*-value	Depressive (*n* = 950)	*p*-value	Insomnia (*n* = 1,234)	*p*-value
Age		66.11 ± 7.51	68.42 ± 9.83	^***^	69.01 ± 9.36	^***^	67.89 ± 9.49	^***^
Gender	Male	564 (38.4)	382 (41.5)	ns	376 (39.7)	ns	492 (39.9)	ns
	Female	875 (61.6)	539 (58.5)		571 (60.3)		741 (60.1)	
Marital status	Unmarried	18 (1.3)	14 (1.5)	ns	15 (1.6)	ns	25 (2.0)	^*^
	Married	1,238 (86.9)	738 (79.9)		757 (79.8)		993 (80.5)	
	Divorced	12 (0.8)	5 (0.5)		8 (0.8)		10 (0.8)	
	Widowed	156 (11)	167 (18.1)		169 (17.8)		206 (16.7)	
Education level	Primary or below	961 (67.6)	701 (75.9)	^**^	746 (78.8)	^**^	920 (74.7)	^**^
	Junior high	336 (23.6)	161 (17.4)		139 (14.7)		215 (17.5)	
	High school or above	124 (8.7)	62 (6.7)		62 (6.5)		97 (7.9)	
Occupation	Farmer/Herder/ Fisherman	1,272 (91.1)	846 (92.7)	^***^	870 (93.2)	^***^	1,110 (91.4)	^*^
	Government/ Institution	43 (3.1)	26 (2.8)		34 (3.6)		39 (3.2)	
	Self-employed	21 (1.5)	12 (1.3)		5 (0.5)		20 (1.6)	
	Enterprise employee	20 (1.40)	12 (1.3)		9 (1.0)		23 (1.9)	
	Unemployed	10 (0.7)	4 (0.4)		7 (0.8)		5 (0.4)	
	Other	30 (2.2)	13 (1.4)		8 (0.9)		18 (1.5)	
Poverty	Yes	58 (4.2)	43 (4.6)	ns	43 (4.5)	ns	67 (5.4)	ns
	No	1,366 (95.8)	881 (95.4)		907 (95.5)		1,167 (94.6)	
Smoking status	Current	202 (14.2)	140 (15.2)	ns	114 (12.1)	ns	167 (13.6)	ns
	Never	1,194 (84.1)	759 (82.2)		807 (85.4)		1.025 (83.7)	
	Former	24 (1.7)	24 (2.6)		24 (2.5)		33 (2.7)	
Alcohol use	Current	228 (16.1)	155 (16.8)	^*^	140 (14.8)	^*^	233 (19.0)	^*^
	Never	1,170 (82.4)	754 (81.7)		793 (83.7)		970 (78.9)	
	Former	22 (1.5)	14 (1.5)		14 (1.5)		26 (2.1)	
Exercise frequency	>2 times/week	766 (60.9)	468 (59.2)	^**^	518 (58.3)	^**^	581 (52.2)	^**^
	1–2 times/week	214 (17)	116 (14.7)		153 (17.2)		231 (20.8)	
	1–3 times/month	107 (8.5)	90 (11.4)		77 (8.7)		106 (9.5)	
	< 1 time/month	170 (13.5)	117 (14.8)		141 (15.9)		195 (17.5)	
Sweet food intake	Frequently	54 (3.8)	19 (2.1)	ns	29 (3.1)	ns	48 (3.9)	ns
	Sometimes	222 (15.7)	152 (16.5)		60 (6.3)		222 (18.1)	
	Occasionally	648 (45.8)	453 (49.1)		347 (36.7)		578 (47.0)	
	Never	490 (34.7)	299 (32.4)		509 (53.9)		381 (31.0)	
Chronic Disease	Yes	1,224 (86)	345 (37.3)	ns	293 (30.8)	^**^	497 (40.3)	ns
	No	200 (14)	579 (62.7)		657 (69.2)		737 (59.7)	
BMI		24.49 ± 7.96	23.98 ± 6.45	ns	24.61 ± 15.76	ns	24.55 ± 13.41	ns
Spousal Relationship	Very Good	693 (56.7)	408 (56.2)	^***^	411 (55.8)	^***^	595 (62.5)	^***^
	Fair	448 (36.7)	254 (35.0)		250 (33.9)		284 (29.8)	
	Poor	81 (6.6)	64 (8.8)		76 (10.3)		73 (7.7)	

*P*-values were calculated by independent samples *t*-tests (for continuous variables, presented as mean ± SD) and chi-square tests [for categorical variables, presented as *n* (%)], respectively, to compare each clinical group (depressive, anxious, insomnia) with the healthy control group. All tests were two-sided, and *p* < 0.05 was considered statistically significant. Unmarried participants (including single, divorced, and widowed individuals) were treated as an independent stratum in all analyses, with missing values recorded for inapplicable spouse-related social relationship factors, without imputation or exclusion to preserve the full sample (*n* = 4,180). ^*^p < 0.05, ^**^p < 0.01, ^***^p < 0.001. ns, not significant.

Comparative analyses revealed significant differences between the groups. Participants who screened positive for anxiety or depressive symptoms were significantly older and had lower educational attainment compared to the healthy control group—defined as individuals whose scores were below the respective clinical thresholds for anxiety, depressive symptoms, and insomnia (*p* < 0.01 for all comparisons). Moreover, the proportion of individuals with anxiety or depressive symptoms who are Farmers/Herders/Fishermen was significantly higher (*p* < 0.001). For insomnia, significant differences were observed across various sociodemographic and health-related factors, including age, marital status, educational level, occupation, alcohol habits, frequency of physical activity, spousal relationship (*p* < 0.05 for all).

### Univariate and multivariate associations with mental health outcomes

Before reporting the associations of individual variables, we first evaluated the overall performance of three multivariate logistic regression models (see Supplementary Table S2). Model fit was assessed using the Hosmer–Lemeshow goodness-of-fit test, which was non-significant for all models, indicating no lack of fit: anxiety symptoms (χ^2^ = 8.96, *p* = 0.34), depression symptoms (χ^2^ = 9.74, *p* = 0.28), and insomnia symptoms (χ^2^ = 10.52, *p* = 0.23). The proportion of variance explained, as indicated by Nagelkerke *R*^2^, was 0.25 for depression, 0.22 for anxiety, and 0.28 for insomnia. Model discrimination was evaluated using the area under the ROC curve (AUC), with all models showing values significantly >0.5: depression AUC = 0.74 (95% CI: 0.71–0.77), anxiety AUC = 0.72 (95% CI: 0.69–0.75), and insomnia AUC = 0.76 (95% CI: 0.73–0.79), demonstrating moderate and acceptable predictive performance.

We then evaluated regression assumptions. Linearity of continuous variables (age, BMI) in the logit was assessed using the Box–Tidwell test, with all variables meeting the linearity assumption (*p* > 0.05, Supplementary Table S5). The study design ensured independence of observations, and the sample size was sufficient, with at least 10 outcome events per predictor variable. Bivariate correlations among predictor variables were examined using Pearson correlation for continuous variables and Spearman's rank correlation for categorical variables; no correlation exceeded 0.5, indicating acceptable independence (Supplementary Table S3). All predictor variables were also assessed for multicollinearity, with VIF values ranging from 1.05 to 1.20 and tolerances above 0.83, confirming no significant multicollinearity (Supplementary Table S4).

Having confirmed the overall adequacy of the models, we next explored the associations of individual variables with anxiety, depression, and insomnia using univariate logistic regression (Table 2). Key determinants included lower education level for all three conditions. Poor spousal relationships were significantly linked to higher odds of both anxiety (OR: 1.40) and depression (OR: 1.77). Moderate physical activity (1–2 times/week) was associated with higher odds of insomnia (OR: 1.40). Notably, the absence of chronic disease was strongly associated with a reduced risk of insomnia (OR: 0.84) but correspondingly increased the odds of depression (OR: 1.45) in univariate analysis.

**Table 2:** Univariate logistic regression analysis for factors associated with anxiety, depression, and insomnia.

Variable	Anxiety_OR (95% CI)	*p*-value	Depressive_OR (95% CI)	*p*-value	Insomnia_OR (95% CI)	*p*-value
Age	1.01 (1.00–1.02)	^**^	1.02 (1.01–1.03)	ns	1.01 (1.00–1.01)	ns
Gender (Female vs. Male)	1.01 (0.87–1.17)	ns	1.11 (0.96–1.29)	ns	1.11 (0.97–1.27)	ns
Marital status (Ref: Unmarried)						
Married	1.02 (0.58–1.92)	ns	0.97 (0.56–1.79)	ns	0.67 (0.41–1.12)	ns
Divorced	0.79 (0.23–2.33)	ns	1.32 (0.47–3.53)	ns	0.88 (0.35–2.17)	ns
Widowed	1.43 (0.79–2.74)	ns	1.33 (0.75–2.50)	ns	0.86 (0.51–1.46)	ns
Education (Ref: Primary or below)						
Junior high	0.77 (0.63–0.92)	^**^	0.59 (0.48–0.72)	^**^	0.77 (0.65–0.91)	^**^
High school or above	0.67 (0.50–0.89)	^**^	0.62 (0.46–0.82)	ns	0.82 (0.64–1.04)	ns
Occupation (Ref: Farmer/Herder/Fisherman)						
Government/Institution	0.70 (0.45–1.06)	ns	0.95 (0.63–1.38)	ns	0.81 (0.56–1.17)	ns
Self-employed	0.77 (0.39–1.41)	ns	0.27 (0.10–0.62)	ns	1.05 (0.61–1.77)	ns
Enterprise employee	0.59 (0.30–1.06)	ns	0.41 (0.19–0.78)	ns	0.94 (0.56–1.50)	ns
Student	0.58 (0.17–1.52)	ns	1.13 (0.44–2.57)	ns	0.53 (0.18–1.29)	ns
Unemployed	0.64 (0.34–1.13)	ns	0.35 (0.16–0.69)	ns	0.66 (0.38–1.10)	ns
Poverty (No vs. Yes)	0.86 (0.60–1.24)	ns	0.88 (0.61–1.29)	^*^	0.66 (0.48–0.91)	^*^
Smoking status (Ref: Current)						
Never	1.00 (0.82–1.23)	ns	1.40 (1.13–1.74)	ns	1.19 (0.98–1.44)	ns
Former	1.37 (0.81–2.24)	ns	1.77 (1.04–2.92)	^*^	1.74 (1.08–2.77)	^*^
Alcohol use (Ref: Current)						
Never	1.11 (0.92–1.35)	ns	1.34 (1.10–1.65)	ns	0.90 (0.76–1.07)	ns
Former	0.99 (0.52–1.78)	ns	1.13 (0.59–2.03)	ns	1.37 (0.81–2.27)	ns
Exercise frequency (Ref: ≥3 times/week)						
1–2 times/week	0.74 (0.59–0.92)	^**^	0.91 (0.74–1.12)	^***^	1.39 (1.15–1.67)	^***^
1–3 times/month	1.24 (0.95–1.61)	ns	0.89 (0.67–1.16)	ns	1.17 (0.91–1.50)	ns
< 1 time/month	0.92 (0.73–1.16)	ns	1.03 (0.83–1.28)	^***^	1.40 (1.15–1.71)	^***^
Sweet food intake (Ref: Frequently)						
Sometimes	2.10 (1.28–3.61)	^**^	0.42 (0.26–0.69)	ns	1.10 (0.76–1.62)	ns
Occasionally	2.10 (1.32–3.55)	^**^	0.91 (0.61–1.41)	ns	0.90 (0.64–1.30)	ns
Never	1.83 (1.14–3.11)	^*^	2.32 (1.55–3.60)	ns	0.78 (0.54–1.12)	ns
Chronic disease (No vs. Yes)	1.00 (0.86–1.17)	ns	1.45 (1.24–1.70)	^*^	0.84 (0.73–0.96)	^*^
BMI	0.99 (0.97–1.00)	ns	1.00 (0.99–1.00)	ns	1.00 (0.99–1.00)	ns
Spousal relationship (Ref: Very Good)						
Fair	1.19 (0.99–1.41)	ns	1.15 (0.96–1.37)	^*^	0.83 (0.71–0.98)	^*^
Poor	1.40 (1.03–1.90)	^*^	1.77 (1.32–2.36)	ns	1.02 (0.76–1.36)	ns

OR, odds ratio; CI, confidence interval.

This table presents unadjusted (crude) ORs from univariate logistic regression analyses, reflecting the bivariate associations between each predictor variable and the outcomes (anxiety, depression, insomnia) without controlling for other covariates. Spousal relationship quality and spouse migrant work status were assessed only among participants who were currently married or cohabiting. Unmarried participants (including single, divorced, and widowed individuals) were treated as an independent stratum, with missing values recorded for spouse-related variables without imputation, preserving the full sample (n=4,180). No equivalent measures were available for non-married participants. Statistical significance is indicated by superscripted asterisks: ^*^p < 0.05; ^**^p < 0.01; ^***^p < 0.001. “ns” denotes non-significant differences (*p* ≥ 0.05).

Variables showing significant associations in univariate analyses were subsequently included in multivariate logistic regression models to identify independent factors associated with each condition (Table 3). The results highlighted distinct risk profiles for each disorder.

**Table 3:** Multivariate Logistic Regression Analysis of Factors Associated with Anxiety, Depression, and Insomnia.

Variable	Anxiety_OR (95% CI)	*p*-value	Depressive_OR (95% CI)	*p*-value	Insomnia_OR (95% CI)	*p*-value
Age	0.99 (0.98–1.00)	ns	1.02 (1.01–1.03)	^***^	1.00 (0.99–1.01)	ns
Gender						
Female (vs. Male)	0.97 (0.77–1.24)	ns	1.05 (0.82–1.34)	ns	1.33 (1.06–1.67)	^*^
Marital status (Ref: Unmarried)						
Married	214,654.10 (NA-NA)	ns	158,243.60 (NA-NA)	ns	186,997.47 (NA-NA)	ns
Divorced	1.05 (0.08–14.21)	ns	1.65 (0.16–20.28)	ns	157,489.06 (NA-NA)	ns
Widowed	192,505.76 (NA-NA)	ns	260,959.46 (NA-NA)	ns	320,643.24 (NA-NA)	ns
Education level (Ref: Primary or below)						
Junior high school	0.76 (0.59–0.98)	^*^	0.79 (0.60–1.04)	ns	0.78 (0.61–0.98)	^*^
High school or above	0.82 (0.55–1.20)	ns	0.80 (0.52–1.21)	ns	0.86 (0.59–1.22)	ns
Occupation (Ref: Farmer/Herder/Fisherman)						
Government/Institution	0.85 (0.48–1.44)	ns	1.03 (0.70–1.79)	ns	0.94 (0.57–1.54)	ns
Self–employed	0.89 (0.41–1.76)	ns	0.44 (0.15–1.05)	ns	1.46 (0.77–2.65)	ns
Enterprise employee	0.72 (0.45–1.37)	ns	0.68 (0.41–1.38)	ns	1.42 (0.81–2.43)	ns
Unemployed	0.84 (0.19–2.68)	ns	1.23 (0.33–3.75)	ns	0.15 (0.01–0.75)	ns
Other	0.72 (0.35–1.36)	ns	0.41 (0.16–0.86)	^*^	0.92 (0.49–1.62)	ns
Poverty (No vs. Yes)	0.66 (0.40–1.14)	ns	0.77 (0.44–1.39)	ns	0.83 (0.50–1.41)	ns
Smoking status (Ref: Current)						
Never	1.04 (0.78–1.41)	ns	1.19 (0.87–1.65)	ns	1.17 (0.89–1.55)	ns
Former	1.61 (0.80–3.11)	ns	1.53 (0.92–3.11)	ns	0.82 (0.39–1.62)	ns
Alcohol use (Ref: Current)						
Never	1.01 (0.76–1.34)	ns	1.18 (0.87–1.61)	ns	0.60 (0.47–0.79)	^***^
Former	0.99 (0.63–2.07)	ns	1.31 (0.56–2.84)	ns	1.21 (0.61–2.36)	ns
Exercise frequency (Ref: ≥3 times/week)						
1–2 times/week	0.69 (0.53–0.90)	^**^	0.85 (0.65–1.11)	ns	1.47 (1.17–1.85)	^***^
1–3 times/month	1.25 (0.91–1.68)	ns	0.94 (0.66–1.31)	ns	1.29 (0.95–1.73)	ns
< 1 time/month	1.07 (0.81–1.40)	ns	0.97 (0.73–1.29)	ns	1.36 (1.05–1.76)	^*^
Sweet food intake (Ref: Frequently)						
Sometimes	1.77 (0.99–3.39)	ns	0.41 (0.23–0.77)	^**^	1.15 (0.74–1.82)	ns
Occasionally	2.05 (1.18–3.82)	^*^	0.97 (0.59–1.69)	ns	0.73 (0.48–1.13)	ns
Never	1.99 (1.14–3.73)	^*^	2.32 (1.40–4.02)	^**^	0.59 (0.38–0.91)	^*^
Chronic disease (No vs. Yes)	1.10 (0.90–1.34)	ns	1.57 (1.28–1.94)	^***^	0.80 (0.66–0.95)	^*^
BMI	0.98 (0.96–1.01)	ns	0.99 (0.97–1.02)	ns	1.00 (0.97–1.02)	ns
Spousal relationship (Ref: Very Good)						
Fair	1.21 (0.98–1.48)	ns	0.98 (0.79–1.22)	ns	0.64 (0.52–0.78)	^***^
Poor	1.55 (1.11–2.16)	^**^	1.09 (0.76–1.55)	ns	0.98 (0.70–1.37)	ns

OR, Odds Ratio; CI, Confidence Interval; Ref, Reference group.

This table presents adjusted odds ratios (ORs) from multivariate logistic regression analyses, reflecting the independent associations between each predictor variable and the outcomes (anxiety, depression, insomnia) after controlling for all measured covariates (age, gender, educational level, occupation, poverty status, smoking status, alcohol use, exercise frequency, BMI, chronic disease status, and spousal relationship quality). Non-married participants (including single, divorced, and widowed individuals) were included as an independent stratified category. Spousal relationship quality was assessed only among participants with a current spouse; for non-married participants, this variable was recorded as NA. Marital status was included as a covariate to control for confounding effects. Notably, NA in the “Married” and “Widowed” categories indicates unreliable OR estimates due to model convergence issues. No multicollinearity was observed among predictors (variance inflation factor [VIF] < 1.5). Statistical significance is indicated by superscripted asterisks: ^*^p < 0.05; ^**^p < 0.01; ^***^p < 0.001. “ns” denotes non-significant differences (*p* ≥ 0.05).

For anxiety symptoms, spousal relationship quality emerged as a predominant independent factor. Among participants with a current spouse or cohabiting partner, a self-reported “poor” relationship was associated with a significantly increased risk (OR: 1.55; 95% CI: 1.11–2.16) relative to a “very good” relationship. Unmarried participants—including those who were single, divorced, or widowed—were treated as an independent stratum, with missing values recorded for spouse-related variables without imputation, thereby preserving the full sample. Engaging in physical activity 1–2 times per week was associated with a lower risk (OR: 0.69; 95% CI: 0.53–0.90) compared to exercising ≥3 times per week. Interestingly, never consuming sweet food was independently associated with higher odds of anxiety (OR: 1.99; 95% CI: 1.14–3.73) relative to frequent consumption.

For depressive symptoms, the absence of a chronic disease remained the strongest associated factor (OR: 1.57; 95% CI: 1.28–1.94). Increasing age was also a significant independent risk factor (OR: 1.02 per year; 95% CI: 1.01–1.03). A non-linear association was observed for sweet food intake: compared to frequent consumption, both “sometimes” (OR: 0.41; 95% CI: 0.23–0.77) and “never” (OR: 2.32; 95% CI: 1.40–4.02) consumption were independently associated with an increased risk of depression.

For insomnia, female gender emerged as an independent risk factor (OR: 1.33; 95% CI: 1.06–1.67). Lower frequencies of physical activity were also independently associated with increased risk (1–2 times/week: OR: 1.47, 95% CI: 1.17–1.85; < 1 time/month: OR: 1.36, 95% CI: 1.05–1.76). Protective factors for insomnia included never drinking alcohol (OR: 0.60; 95% CI: 0.47–0.79), the absence of chronic disease (OR: 0.80; 95% CI: 0.66–0.95), and a “fair” spousal relationship (OR: 0.64; 95% CI: 0.52–0.78).

### Development and presentation of clinical prediction nomograms

**Figure 1:**
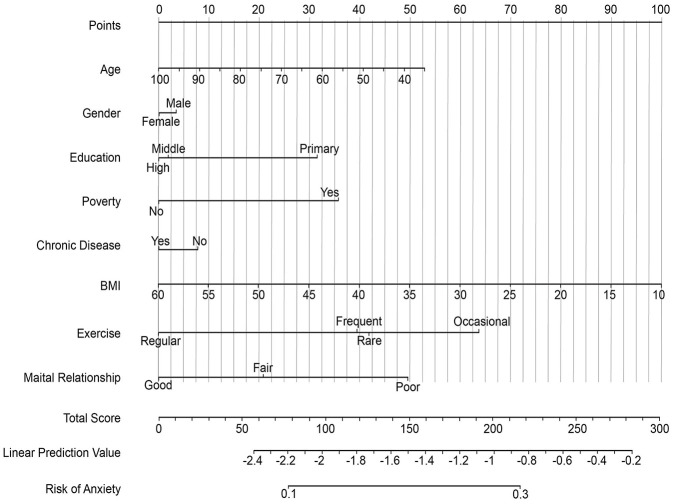
Nomogram for predicting the risk of anxiety in rural Chinese older adults (≥55 years). Each patient's characteristics across all predictive variables (Age, Gender, Education, Poverty, Chronic Disease, BMI, Exercise, Marital Relationship) are converted into point values. These points are summed to generate a Total Score (upper row). The Total Score is linearly transformed into a Linear Prediction Value (lower row, log-odds scale), which is then mapped to the final predicted probability of anxiety (bottom row). Variable labels and spacing have been adjusted for clarity to prevent overlap.

**Figure 2:**
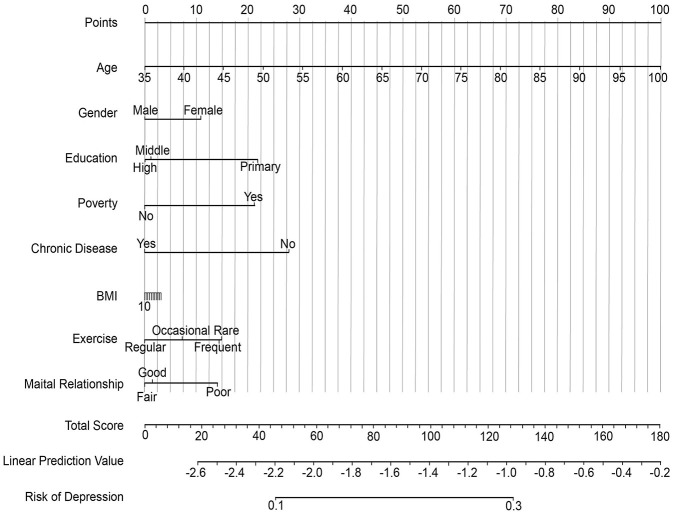
Nomogram for predicting the risk of depression in rural Chinese older adults (≥55 years). Patient characteristics across all predictive variables (Age, Gender, Education, Poverty, Chronic Disease, BMI, Exercise, Marital Relationship) are assigned point values. The points are summed to produce a Total Score (upper row), which is linearly transformed to a Linear Prediction Value (lower row, log-odds scale) and mapped to the final predicted probability of depression (bottom row). Variable labels have been adjusted for readability.

**Figure 3:**
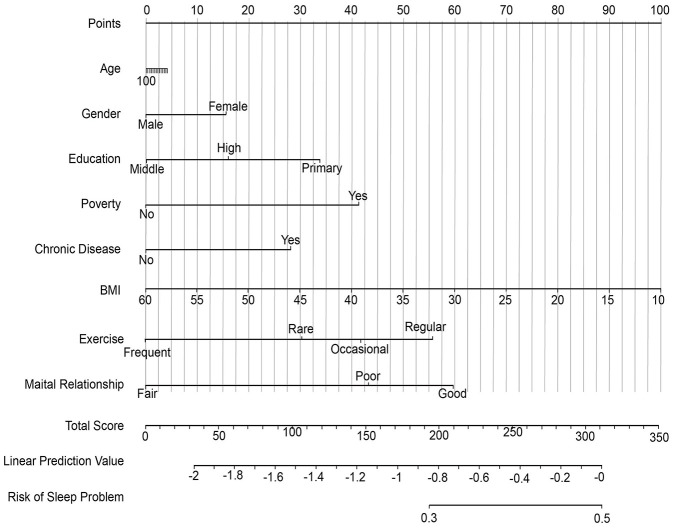
Nomogram for predicting the risk of sleep problems in rural Chinese older adults (≥55 years). Point values assigned to each predictive variable (Age, Gender, Education, Poverty, Chronic Disease, BMI, Exercise, Marital Relationship) are summed to obtain a Total Score (upper row). This Total Score is linearly transformed into a Linear Prediction Value (lower row, log-odds scale) and used to derive the final predicted probability of sleep problems (bottom row). Variable labels and spacing have been adjusted to avoid overlap and improve readability.

Based on the final multivariate models, we developed three nomograms for the individualized prediction of anxiety, depression, and insomnia (Figures 1–3). These nomograms integrated the identified independent predictors, including age, gender, education, poverty status, chronic disease, BMI, exercise frequency, and spousal relationship quality, with point assignments scaled proportionally to their respective regression coefficients. For unmarried participants, spouse-related indicators were treated as missing values; thus, the corresponding results primarily reflect the married or cohabiting subgroup. In general, female gender, lower educational attainment, poverty, infrequent exercise, and poor spousal relationships were associated with higher point scores across all models, whereas unique patterns such as older age in depression and younger age in insomnia further distinguish condition-specific risks. To use these nomograms, clinicians can match a patient's characteristics to the corresponding variable axes, sum the total points, and then project the total score onto the bottom risk axis to obtain the estimated probability of developing each condition. Internal validation was performed using 1,000 bootstrap resamples. After optimism correction, the AUC values were 0.71 (95% CI: 0.68–0.74) for anxiety, 0.73 (95% CI: 0.70–0.76) for depression, and 0.75 (95% CI: 0.72–0.78) for insomnia, indicating favorable discriminatory ability with minimal overfitting. Calibration curves further confirmed excellent consistency between predicted and observed probabilities, with slopes close to 1.0 and intercepts near 0. Collectively, these findings support the reasonable internal validity of the nomograms in the study cohort, although external validation in independent rural populations is still warranted before wider clinical application (Supplementary Table S6).

## Discussion

Our study delineates distinct yet overlapping risk architectures for geriatric depression, anxiety, and insomnia within a rural Chinese cohort, revealing the necessity of a differentiated conceptual framework for these conditions. This approach is consistent with, and extends, findings from other studies that have employed nomograms to model disease risk in specific populations (22). For instance, similar to our work, a nomogram model for colorectal cancer in a rural area identified lifestyle factors (e.g., smoking, diet) as key predictors, highlighting the utility of such models in capturing population-specific risk profiles. While shared socioeconomic vulnerabilities—particularly lower educational attainment and poverty—formed a common baseline risk in our cohort, mirroring the social determinants of health often observed in rural communities, the predominant independent predictors revealed strikingly divergent pathways for each mental health condition (23, 24).

The most notable finding was the robust association between the absence of a diagnosed chronic disease and increased depressive symptoms, which appears counterintuitive compared to studies in other populations (25). One possible explanation is the unique context of rural China, where limited mental health literacy and strong stigma may lead older adults to express psychological distress primarily through somatic complaints rather than seeking formal diagnoses (26). Individuals without chronic disease diagnoses may therefore represent a subgroup whose depressive symptoms are not recognized or treated, while those with chronic conditions may receive more frequent medical attention, social support, and opportunities for early mental health intervention. This counterintuitive association can be further understood from four culture-specific perspectives in rural China. First, older adults without chronic diseases often underreport psychological distress due to low mental health literacy and the strong stigma surrounding mental illness, leading to depressive symptoms being somatized as vague physical discomfort rather than recognized as an emotional disorder (20, 27). Second, older adults with chronic diseases undergo regular medical follow-ups, medication management, and family monitoring, which provides caregivers and clinicians with more opportunities to detect emotional abnormalities and offer early support. In contrast, those without a diagnosed illness rarely seek medical care and remain invisible within routine health monitoring systems (28, 29). Third, the absence of chronic disease may lead to a silent psychological burden associated with fear of future illness, lack of health awareness, or lack of social attention, gradually triggering depressive symptoms that traditional physical health indicators cannot capture (30). Fourth, in rural communities with limited mental health services, physically healthy older adults are less likely to have their depressive symptoms identified or managed, resulting in higher scores and a risk that appears higher than that of chronic disease patients who receive more comprehensive care (10). These factors collectively explain why the absence of chronic disease has become an independent risk factor for depressive symptoms in this rural older adult cohort, reflecting detection bias, somatization, and insufficient mental health resources, rather than a true causal effect of physical health on emotional well-being. This finding highlights the importance of culturally and contextually tailored screening strategies to identify depression among older adults who appear physically healthy (31).

For anxiety symptoms, spousal relationship quality emerged as the dominant factor, highlighting the paramount importance of intimate social bonds in buffering age-related uncertainties. This aligns with cognitive models that emphasize the role of interpersonal factors in emotional disorders and finds parallels in intervention studies (32). For instance, the observed importance of relationship quality in anxiety resonates with the focus of Interpersonal Therapy (IPT) in addressing relational issues to improve mental health outcomes (33). It is important to note that the social relationship variables included in our study—spousal relationship quality and spouse migrant work status—were assessed only among participants who were currently married or cohabiting. Unmarried participants, including those who were single, divorced, or widowed, were treated as an independent stratified group, with missing values recorded for spouse-related variables without imputation, thereby preserving the full sample. Conversely, insomnia was characterized primarily by behavioral and physiological determinants, with reduced exercise frequency and female gender constituting key risk factors. The protective effect of never drinking alcohol for insomnia is readily interpretable through a physiological lens, as alcohol can disrupt sleep architecture (34).

These findings were further refined by the non-linear relationships observed. The superior protective effect of exercising 1–2 weekly sessions against anxiety, compared to higher frequencies, suggests an optimal balance between physiological benefit and avoiding exercise as a potential stressor in this older adult population. This nuanced pattern may not be captured in risk models that treat exercise as a binary exposure (35). The complex associations of sweet food intake with depression and anxiety invite multifactorial explanations. The U-shaped relationship with depression suggests that while complete avoidance may indicate restrictive eating patterns associated with psychological distress, occasional consumption might serve as an effective mood regulation strategy in this cultural context. These complex relationships underscore the value of using non-linear terms or stratified categories in nomogram development to improve predictive accuracy, a approach also adopted in a sophisticated nomogram for intrahepatic cholangiocarcinoma which integrated multiple clinical variables for superior prognosis prediction (36, 37).

The development of condition-specific nomograms represents a crucial advancement in translating these epidemiological findings into practical tools for frontline care in resource-limited settings. Our visual models enable rapid risk stratification by primary care workers, facilitating early identification of high-risk individuals who rarely seek specialized mental healthcare (38). This aligns with the broader application of nomograms in various medical fields to support clinical decision-making. More importantly, the distinct risk profiles we identified facilitate precisely targeted interventions (39). For example, patients with depression risk driven by absence of chronic disease warrant assessment for somatization, while those with anxiety risk linked to poor spousal relationships would benefit more from relationship-focused interventions than generic recommendations. This tailored approach is supported by evidence from mental health interventions suggesting that targeting specific risk factors can be more effective than generic approaches (40).

Beyond statistical significance, we further considered the clinical relevance of our findings, as statistically significant associations do not always translate into practical value in primary care settings. Our results, based on a large rural cohort (*n* = 4,180), offer actionable insights for targeted mental health interventions due to their clear clinical relevance: For anxiety, poor spousal relationships (OR: 1.55) played a major role, while moderate physical activity (1–2 times weekly; OR: 0.69) offered protective effects, holding direct clinical significance. Adjusting interpersonal interactions according to rural cultural customs may address underlying causes of anxiety. Furthermore, recommending moderate exercise (rather than high-intensity activity) is more feasible for older adults with limited mobility, ensuring better adherence and clinical utility. For depression, significant associations with the absence of chronic diseases (OR: 1.57) and older age (OR: 1.02 per year) highlight the necessity of screening for depressive symptoms among rural older adults aged 70 and above without diagnosed physical illnesses. As clinical attention often focuses on those with chronic conditions, this group is frequently overlooked in routine care. For insomnia (prevalence: 29.5%), female gender (OR: 1.33) and lower exercise frequency showed significant associations, providing clear targets for lifestyle interventions. Primary healthcare providers in rural settings can promote structured low-intensity physical activities, such as morning walks or Tai Chi, which are low-cost and easy to implement. The protective effect of abstaining from alcohol (OR: 0.60) also supports health education campaigns on alcohol's disruptive impact on sleep structure, aligning with rural older adults' preference for non-pharmacological interventions.

Our study benefits from its foundation in the community-based Taierzhuang Aging Cohort, which ensures representative sampling. The simultaneous evaluation of three mental health conditions within a substantial sample, coupled with the development of clinically applicable nomograms, represents a significant methodological advancement over studies focusing on single outcomes. Nevertheless, several limitations should be acknowledged to appropriately contextualize our findings. First, despite the statistical significance of the findings in this study, certain effects—such as the relationship between the absence of chronic disease and depression—may not hold substantial clinical significance in practice. When interpreting such results, particularly in studies based on large samples, careful consideration must be given to whether small effect sizes truly impact patient management. Second, the cross-sectional design precludes causal inference, and self-reported data may be susceptible to recall and social desirability biases. Furthermore, while the nomograms demonstrated good internal performance, external validation in other rural Chinese populations is necessary before widespread implementation, a step that was crucial in establishing the robustness of other published nomograms.

Building upon these findings, we propose key directions for future research. The planned expansion of the Taierzhuang Cohort provides an ideal platform for prospective studies to establish causal pathways. External validation of our nomograms is essential for generalizability. Most importantly, the identified risk profiles should inform the development of targeted interventions—such as relationship counseling for anxiety prevention and structured exercise programs for sleep health—that can translate our findings into tangible improvements in rural older adult mental wellbeing. Such interventions could draw on established evidence-based psychotherapeutic approaches like CBT and IPT, while carefully considering their adaptation to the rural older adult context and potential implementation challenges.

## Conclusion

This study establishes distinct risk profiles for geriatric mental health conditions in rural China, with depression linked to somatic expression, anxiety to relational factors, and insomnia to behavioral determinants. We developed and validated practical nomograms (Figures 1–3) that effectively translate these differential risk patterns into clinically applicable tools for individualized risk assessment and targeted intervention in primary care settings.

## Data Availability

The raw data supporting the conclusions of this article will be made available by the authors, upon reasonable request.
