# Correction: Differential risk profiles for geriatric depression, anxiety, and sleep disturbances in rural China: insights from the Taierzhuang cohort

**DOI:** 10.3389/fpubh.2026.1888662

**Published:** 2026-06-12

**Authors:** Renfeng Zhang, Qian Yu, Yunshan Wang, Xiaoyi Zhao, Shuyi Yu, Zhongyu Dong, Jinghai Hou, Xingguo Qin, Ming Li, Mo Wang

**Affiliations:** 1 Department of Clinical Laboratory, Shandong Provincial Hospital Affiliated to Shandong First Medical University, Jinan, China; 2 People's Hospital of Taierzhuang District, Zaozhuang, China; 3 Department of Neurology, People's Hospital of Taierzhuang District, Zaozhuang, China; 4 Department of Vascular Surgery, Shandong Provincial Hospital Affiliated to Shandong First Medical University, Jinan, Shandong, China

**Keywords:** anxiety, cohort, depression, geriatric mental health, public health

Affiliation for Xingguo Qin was erroneously given as “People's Hospital of Taierzhuang District, Zaozhuang, China.” The correct affiliation is “Department of Neurology, People's Hospital of Taierzhuang District, Zaozhuang, China.”

Affiliation for Mo Wang was erroneously given as “Department of Clinical Laboratory, Shandong Provincial Hospital Affiliated to Shandong First Medical University, Jinan, China.” The correct affiliation is “Department of Vascular Surgery, Shandong Provincial Hospital Affiliated to Shandong First Medical University, Jinan, Shandong, China.”

Authors Renfeng Zhang and Qian Yu were erroneously omitted as equal contributing authors. “These authors have contributed equally to this work and share first authorship” has been added as a footnote for these authors.

The original version of this article has been updated.

