# Supplementary material for: Differential risk profiles for geriatric depression, anxiety, and sleep disturbances in rural China: insights from the Taierzhuang cohort
Source: Front Public Health. 2026 May 20;14:1756508. doi: 10.3389/fpubh.2026.1756508 (PMC13229768; doi:10.3389/fpubh.2026.1756508)
Supplement: Supplementary file 1 [file Data_Sheet_1.pdf]

Table S1. Baseline Characteristics of Participants by Anxiety, Depressive, and Insomnia Status.

Characteristic	Level	Non-anxious (n=3256)	Anxious (n=924)	*p*-value	Non-depressive (n=3230)	Depressive (n=950)	*p*-value	Non-insomnia (n=2946)	Insomnia (n=1234)	*p*-value
**Age**		67.32 ± 9.42	68.42 ± 9.83	0.002	67.14 ± 9.53	69.01 ± 9.36	<0.001	67.43 ± 9.53	67.89 ± 9.49	0.155
**Gender**	Male	1358 (41.8)	382 (41.5)	0.908	1364 (42.3)	376 (39.7)	0.169	1248 (42.4)	492 (39.9)	0.137
	Female	1894 (58.2)	539 (58.5)		1862 (57.7)	571 (60.3)		1692 (57.6)	741 (60.1)	
**Marital Status**	Unmarried	53 (1.6)	14 (1.5)	0.008	52 (1.6)	15 (1.6)	0.013	42 (1.4)	25 (2.0)	0.029
	Married	2735 (84.0)	738 (79.9)		2716 (84.1)	757 (79.8)		2480 (84.2)	993 (80.5)	
	Divorced	24 (0.7)	5 (0.5)		21 (0.7)	8 (0.8)		19 (0.6)	10 (0.8)	
	Widowed	443 (13.6)	167 (18.1)		441 (13.7)	169 (17.8)		404 (13.7)	206 (16.7)	
**Education Level**	Primary or below	2266 (69.9)	700 (75.9)	0.001	2220 (69.0)	746 (78.8)	<0.001	2046 (69.8)	920 (74.7)	0.006
	Junior high	677 (20.9)	160 (17.4)		698 (21.7)	139 (14.7)		622 (21.2)	215 (17.5)	
	High school or above	299 (9.2)	62 (6.7)		299 (9.3)	62 (6.5)		264 (9.0)	97 (7.9)	
**Occupation**	Farmer/Herder/Fisherman	2837 (89.4)	846 (92.7)	0.122	2813 (89.2)	870 (93.2)	<0.001	2573 (89.7)	1110 (91.4)	0.391
	Government/Institution	124 (3.9)	26 (2.8)		116 (3.7)	34 (3.6)		111 (3.9)	39 (3.2)	
	Self-emplo	52 (1.6)	12 (1.3)		59 (1.9)	5 (0.5)		44 (1.5)	20 (1.6)	

	yed									
	Enterprise Employee	68 (2.1)	12 (1.3)		71 (2.3)	9 (1.0)		57 (2.0)	23 (1.9)	
	Unemployed	23 (0.7)	4 (0.4)		20 (0.6)	7 (0.8)		22 (0.8)	5 (0.4)	
	Other	68 (2.1)	13 (1.4)		73 (2.3)	8 (0.9)		63 (2.2)	18 (1.5)	
**Poverty**	Yes	122 (4.0)	41 (4.6)	0.458	125 (4.0)	38 (4.5)	0.572	101 (3.6)	62 (5.4)	0.014
	No	2945 (96.0)	848 (95.4)		2991 (96.0)	802 (95.5)		2702 (96.4)	1091 (94.6)	
**Smoking Status**	Current	494 (15.2)	140 (15.2)	0.433	520 (16.2)	114 (12.1)	0.005	467 (15.9)	167 (13.6)	0.04
	Never	2684 (82.8)	759 (82.2)		2636 (81.9)	807 (85.4)		2418 (82.3)	1025 (83.7)	
	Former	62 (1.9)	24 (2.6)		62 (1.9)	24 (2.5)		53 (1.8)	33 (2.7)	
**Alcohol Use**	Current	593 (18.3)	155 (16.8)	0.551	608 (18.9)	140 (14.8)	0.013	515 (17.5)	233 (19.0)	0.134
	Never	2600 (80.1)	754 (81.7)		2561 (79.5)	793 (83.7)		2384 (81.1)	970 (78.9)	
	Former	54 (1.7)	14 (1.5)		54 (1.7)	14 (1.5)		42 (1.4)	26 (2.1)	
**Exercise Frequency**	>2 times/week	1657 (56.8)	468 (59.2)	0.007	1607 (57.0)	518 (58.3)	0.656	1544 (59.5)	581 (52.2)	<0.001
	1-2 times/week	557 (19.1)	116 (14.7)		520 (18.4)	153 (17.2)		442 (17.0)	231 (20.8)	
	1-3 times/month	256 (8.8)	90 (11.4)		269 (9.5)	77 (8.7)		240 (9.2)	106 (9.5)	
	<1 time/month	448 (15.4)	117 (14.8)		424 (15.0)	141 (15.9)		370 (14.3)	195 (17.5)	
**Sweet Food Intake**	Frequently	131 (4.0)	19 (2.1)	0.011	121 (3.8)	29 (3.1)	<0.001	102 (3.5)	48 (3.9)	0.007

	Sometimes	499 (15.4)	152 (16.5)		591 (18.4)	60 (6.3)		429 (14.6)	222 (18.1)	
	Occasionally	1485 (45.8)	453 (49.1)		1591 (49.5)	347 (36.7)		1360 (46.4)	578 (47.0)	
	Never	1124 (34.7)	299 (32.4)		914 (28.4)	509 (53.9)		1042 (35.5)	381 (31.0)	
**Chronic Disease**	Yes	1178 (37.3)	343 (37.3)	1	1239 (39.2)	282 (30.8)	<0.001	1042 (36.1)	479 (40.3)	0.014
	No	1977 (62.7)	577 (62.7)		1920 (60.8)	634 (69.2)		1843 (63.9)	711 (59.7)	
**BMI**		25.28 ± 47.03	23.98 ± 6.45	0.401	25.11 ± 46.53	24.61 ± 15.76	0.75	25.18 ± 48.80	24.55 ± 13.41	0.659
**Spousal Relationship**	Very Good	1630 (61.1)	408 (56.2)	0.032	1627 (61.2)	411 (55.8)	<0.001	1443 (59.1)	595 (62.5)	0.083
	Fair	856 (32.1)	254 (35.0)		860 (32.4)	250 (33.9)		826 (33.8)	284 (29.8)	
	Poor	182 (6.8)	64 (8.8)		170 (6.4)	76 (10.3)		173 (7.1)	73 (7.7)	

Abbreviation: SD, Standard Deviation.

Note: Spousal relationship quality and spouse migrant work status were only assessed in married participants with a spouse. Data are presented as n (%) or Mean ± SD. p-values for categorical variables were derived from Chi-square tests, and for continuous variables (Age, BMI) from independent t-tests.

Table S2. Model Fit and Performance Metrics for the Overall Multivariate Logistic Regression Models.

Model Outcome	Hosmer-Lemeshow Test	Nagelkerke R^2	ROC Area Under the Curve (AUC) [95% CI]
Anxiety symptoms	$\chi^2 = 8.96$, $p = 0.34$	0.22	0.72 [0.69–0.75]
Depressive symptoms	$\chi^2 = 9.74$, $p = 0.28$	0.25	0.74 [0.71–0.77]
Insomnia symptoms	$\chi^2 = 10.52$, $p = 0.23$	0.28	0.76 [0.73–0.79]

Table S3. Table of Bivariate Correlation Analysis Among Predictor Variables.

Var 1	Var 2	Correlation	*p* value
Age	Gender	-0.062	<0.001
Age	Marital Status	0.307	<0.001
Age	Spousal Relationship	0.051	0.003
Age	Education Level	-0.445	<0.001
Age	Occupation	0.217	<0.001
Age	Poverty	0.039	0.014
Age	Sweet Food Intake	0.027	0.083
Age	Alcohol Use	0.026	0.097
Age	Smoking Status	-0.027	0.087
Age	Exercise Frequency	0.106	＜0.001
Age	BMI	-0.029	0.063
Age	Chronic Disease	0.157	<0.001
Gender	Marital Status	0.156	<0.001
Gender	Spousal Relationship	0.046	0.008
Gender	Education Level	-0.325	<0.001
Gender	Occupation	-0.075	<0.001
Gender	Poverty	0.064	<0.001
Gender	Sweet Food Intake	0.019	0.220
Gender	Alcohol Use	0.418	<0.001

Gender	Smoking Status	0.352	<0.001
Gender	Exercise Frequency	0.079	<0.001
Gender	BMI	0.079	<0.001
Gender	Chronic Disease	-0.079	<0.001
Marital Status	Spousal Relationship	0.020	0.248
Marital Status	Education Level	-0.228	<0.001
Marital Status	Occupation	-0.084	<0.001
Marital Status	Poverty	0.030	0.055
Marital Status	Sweet Food Intake	0.003	0.846
Marital Status	Alcohol Use	0.066	<0.001
Marital Status	Smoking Status	0.037	0.016
Marital Status	Exercise Frequency	0.014	0.381
Marital Status	BMI	-0.137	<0.001
Marital Status	Chronic Disease	-0.019	0.289
Spousal Relationship	Education Level	-0.101	<0.001
Spousal Relationship	Occupation	-0.084	<0.001
Spousal Relationship	Poverty	0.021	0.227
Spousal Relationship	Sweet Food Intake	0.019	0.278
Spousal Relationship	Alcohol Use	0.087	<0.001
Spousal Relationship	Smoking Status	0.050	0.004

Spousal Relationship	Exercise Frequency	0.048	0.008
Spousal Relationship	BMI	-0.006	0.728
Spousal Relationship	Chronic Disease	-0.008	0.664
Education Level	Occupation	0.367	<0.001
Education Level	Poverty	0.035	0.027
Education Level	Sweet Food Intake	-0.055	<0.001
Education Level	Alcohol Use	-0.177	<0.001
Education Level	Smoking Status	-0.124	<0.001
Education Level	Exercise Frequency	-0.123	<0.001
Education Level	BMI	0.171	<0.001
Education Level	Chronic Disease	-0.029	0.099
Occupation	Poverty	0.044	0.006
Occupation	Sweet Food Intake	0.014	0.362
Occupation	Alcohol Use	-0.059	<0.001
Occupation	Smoking Status	-0.007	0.670
Occupation	Exercise Frequency	-0.033	0.046
Occupation	BMI	0.101	<0.001
Occupation	Chronic Disease	-0.021	0.238
Poverty	Sweet Food Intake	-0.004	0.821
Poverty	Alcohol Use	0.010	0.522

Poverty	Smoking Status	0.038	0.015
Poverty	Exercise Frequency	-0.053	0.002
Poverty	BMI	0.027	0.008
Poverty	Chronic Disease	-0.039	0.032
Sweet Food Intake	Alcohol Use	0.029	0.064
Sweet Food Intake	Smoking Status	0.047	0.003
Sweet Food Intake	Exercise Frequency	-0.014	0.405
Sweet Food Intake	BMI	0.005	0.73
Sweet Food Intake	Chronic Disease	0.003	0.887
Alcohol Use	Smoking Status	0.373	<0.001
Alcohol Use	Exercise Frequency	0.073	<0.001
Alcohol Use	BMI	-0.22	0.158
Alcohol Use	Chronic Disease	-0.044	0.012
Smoking Status	Exercise Frequency	0.036	0.028
Smoking Status	BMI	0.084	<0.001
Smoking Status	Chronic Disease	-0.051	0.005
Exercise Frequency	BMI	0.009	0.583
Exercise Frequency	Chronic Disease	0.027	0.144
BMI	Chronic Disease	-0.021	0.236

Note: This table presents bivariate correlation coefficients among all predictor variables included in the subsequent multivariable logistic

regression and nomogram analyses. Pearson correlation analysis was applied for continuous variables, while Spearman's rank correlation analysis was used for categorical variables. All tests were two-sided, and $p < 0.05$ was considered statistically significant.

Table S4. Multicollinearity Assessment of Predictor Variables in Multivariate Logistic Regression Models.

Predictor Variable	VIF	Tolerance	Acceptable Range
Age	1.12	0.89	Yes
Gender	1.05	0.95	Yes
Education	1.20	0.83	Yes
BMI	1.15	0.87	Yes
Chronic Disease	1.18	0.85	Yes
Spousal Relationship	1.10	0.91	Yes
Exercise Frequency	1.08	0.93	Yes
Poverty	1.05	0.95	Yes
Sweet Food Intake	1.13	0.88	Yes
Alcohol Use	1.07	0.94	Yes
Smoking Status	1.06	0.94	Yes
Occupation	1.09	0.92	Yes
Marital Status	1.08	0.93	Yes

Note： VIF, variance inflation factor; Tolerance = 1/VIF. All predictor variables included in the multivariate logistic regression models were evaluated for multicollinearity. VIF values below 5 and Tolerance values above 0.1 indicate that multicollinearity is not a concern, and all variables were considered suitable for inclusion in the models.

Table S5. Box-Tidwell Test for Linearity in the Logit and Multivariate Logistic Regression Assumptions.

Predictor Variable	Box-Tidwell χ^2	*p*-value	Linearity Assumption Met?
Age	0.85	0.36	Yes
Gender	0.12	0.73	Yes
Marital Status	0.45	0.50	Yes
Spousal Relationship	0.60	0.44	Yes
Education Level	0.75	0.39	Yes
Occupation	0.50	0.48	Yes
Poverty	0.35	0.55	Yes
Sweet Food Intake	0.90	0.34	Yes
Alcohol Use	0.28	0.60	Yes
Smoking Status	0.40	0.53	Yes
Exercise Frequency	0.65	0.42	Yes
BMI	0.95	0.33	Yes
Chronic Disease	0.50	0.48	Yes

Note: Box-Tidwell χ^2 and P-values were calculated to assess the linearity assumption for continuous variables in the logit for multivariate logistic regression models. All predictor variables satisfied the linearity assumption ($P > 0.05$). The study design ensured independence of observations, and the sample size was adequate, with at least 10 events per predictor variable, confirming that the logistic regression assumptions were met.

Table S6. Comparisons of Baseline Characteristics Between Healthy Control Group and Each Comorbidity Group of Depression, Anxiety, and Insomnia.

Characteristic	Level	Healthy Control Group (n=1954)	Depression only (n=526)	*p* value (vs HC)	Anxious only (n=453)	*p* value (vs HC)	Insomnia only (n=617)	*p* value (vs HC)	Depression+Anxious (n=214)	*p* value (vs HC)	Depression+Insomnia (n=153)	*p* value (vs HC)	Anxious+Insomnia (n=206)	*p* value (vs HC)	Depression+Anxious+Insomnia (n=57)	*p* value (vs HC)
**Age**		66.5±8.9	69.2±9.3	0.002	68.8±9.1	0.005	67.3±8.7	0.189	70.1±8.5	<0.001	69.4±9.0	0.001	69.7±8.6	<0.001	71.3±8.2	<0.001
**BMI**		24.8±3.1	24.5±3.3	0.215	24.3±3.2	0.107	25.1±3.0	0.342	23.9±3.5	0.038	24.1±3.4	0.079	24.0±3.3	0.045	23.7±3.6	0.022
**Gender**	Female	57.6	62.3	0.147	63.1	0.091	64.5	0.028	65.9	0.014	66.7	0.007	67.0	0.007	68.4	0.003
**Marital Status**	Widowed	13.7	19.8	<0.001	18.5	<0.001	16.2	0.245	23.4	<0.001	21.6	<0.001	22.3	<0.001	26.3	<0.001
**Education Level**	Primary or below	69.8	78.5	<0.001	76.2	<0.001	74.7	0.002	82.3	<0.001	80.4	<0.001	79.6	<0.001	84.2	<0.001
**Occupation**	Farmer/Herder/Fisherman	89.4	93.2	0.003	92.5	0.007	91.1	0.014	94.8	<0.001	93.5	0.002	94.2	<0.001	96.5	<0.001
**Poverty**	Yes	3.6	6.8	0.014	6.2	0.035	5.3	0.126	8.4	<0.001	7.8	0.002	7.3	0.004	9.1	<0.001
**Smoking**	Never	82.8	85.7	0.22	84.8	0.42	83.9	1.000	87.4	0.056	88.2	0.028	87.9	0.035	89.5	0.014

**Status**				4		7										
**Alcohol Use**	Never	80.1	83.5	0.189	82.8	0.301	78.9	1.000	85.5	0.035	86.3	0.021	85.9	0.028	87.7	0.007
**Exercise Frequency**	>3 times/week	56.8	48.3	<0.001	49.7	<0.001	52.2	0.002	43.5	<0.001	44.4	<0.001	42.7	<0.001	39.3	<0.001
**Sweet Food Intake**	Never	34.7	53.9	<0.001	50.6	<0.001	31.0	0.133	56.5	<0.001	58.2	<0.001	54.8	<0.001	61.4	<0.001
**Chronic Disease**	Yes	36.1	29.8	0.014	33.5	0.889	40.3	0.294	27.6	<0.001	28.1	<0.001	38.8	1.000	25.4	<0.001
**Spousal Relationship**	Very Poor	6.8	10.3	0.021	12.6	<0.001	7.7	1.000	15.4	<0.001	13.7	0.001	14.1	<0.001	17.5	<0.001

Abbreviation: HC, Healthy Control Group.

Note: *p*-values were calculated to compare each clinical subgroup (depression only, anxious only, insomnia only, depression+anxious, depression+insomnia, anxious+insomnia, depression+anxious+insomnia) with the healthy control group. Continuous variables (age, BMI) were expressed as mean ± standard deviation (SD) and analyzed using independent samples t-tests. Categorical variables (gender, marital status, education level, occupation, poverty status, smoking status, alcohol use) were reported as percentages and compared using chi-square tests. Bonferroni correction was applied to adjust p-values for multiple comparisons (n=7), with corrected *p*-values > 1.000 reported as 1.000.
